# Independently tunable dual-band perfect absorber based on graphene at mid-infrared frequencies

**DOI:** 10.1038/srep18463

**Published:** 2015-12-22

**Authors:** Yuping Zhang, Tongtong Li, Qi Chen, Huiyun Zhang, John F. O’Hara, Ethan Abele, Antoinette J. Taylor, Hou-Tong Chen, Abul K. Azad

**Affiliations:** 1 Qingdao Key Laboratory of Terahertz Technology, College of Electronic Communication and Physics, Shandong University of Science and Technology, Qingdao, Shandong 266510, China; 2 Center for Integrated Nanotechnologies, Materials Physics and Applications Division, Los Alamos National Laboratory, Los Alamos, NM 87545, USA; 3 Institute of Electronic Engineering, China Academy of Engineering Physics, Mianyang, Sichuan 621999, China; 4 School of Electrical and Computer Engineering, Oklahoma State University, Stillwater, OK 74078, USA

## Abstract

We design a dual-band absorber formed by combining two cross-shaped metallic resonators of different sizes within a super-unit-cell arranged in mirror symmetry. Simulations indicate that absorption efficiencies greater than 99% can be achieved at two different frequencies under normal incidence. We employ a design scheme with graphene integration, which allows independent tuning of individual absorption frequencies by electrostatically changing the Fermi energy of the graphene layer. High absorbance is maintained over a wide incident angle range up to 50 degrees for both TE and TM polarizations. It thus enables a promising way to design electrically tunable absorbers, which may contribute toward the realization of frequency selective detectors for sensing applications.

Metamaterial absorbers^1^ have attracted considerable attention in the past few years because of their promising applications in stealth, wavelength selective emission, sensing, and spectroscopy^2^. A variety of metamaterial-based perfect absorbers have been demonstrated at microwave^3^,^4^, terahertz^5^,^6^,^7^,^8^, infrared^9^,^10^,^11^,^12^, and visible frequencies^13^, operating from a single narrow-band to multiband and broadband employing complex super-unit-cells composed of multiple resonators of different sizes^14^,^15^,^16^,^17^,^18^,^19^,^20^,^21^,^22^. There are many techniques to actively and dynamically control the resonance frequency, including optical illumination^23^,^24^, mechanically adjustment^25^,^26^, fluid filling^27^, electronic charge injection^28^, and temperature variations^29^,^30^. Among them, electrically tuning is highly desired because of the convenience and high switching rate. Recently, there have been some efforts in investigating tunable metamaterial absorbers based on graphene^31^,^32^,^33^. Graphene, consisting of one monolayer of carbon atoms arranged in a honeycomb lattice, offers unique properties such as high optical transparency, flexibility, high electron mobility^34^,^35^, and tunable conductivity^36^,^37^. In particular, the electrostatic control of conductivity makes graphene a promising candidate for designing tunable metamaterials at terahertz and infrared frequencies^36^,^38^,^39^,^40^,^41^,^42^,^43^. Most graphene-based metamaterial absorbers demonstrated single or multiband operation where absorption bands were tuned simultaneously in the same manner. For some applications, such as frequency selective sensing, it may be highly desirable to tune specific absorption bands while keeping others fixed. However, to date there is no extant report on multiband metamaterial absorbers based on graphene that can independently tune absorption bands.

In this paper, we numerically demonstrate, via highly confident numerical simulations, a dual-band metamaterial absorber where the two absorption bands can be independently tuned. This can be accomplished through the application of a voltage bias to modify the conductivity of the graphene layer that has been integrated into the metamaterial absorber super-unit-cell. The metamaterial device offers high absorbance over a wide range of incidence angles, and the independently tunable absorption bands might find applications in fields like tunable sensors and selective thermal emitters.

## Results and Discussions

**Figure 1:**
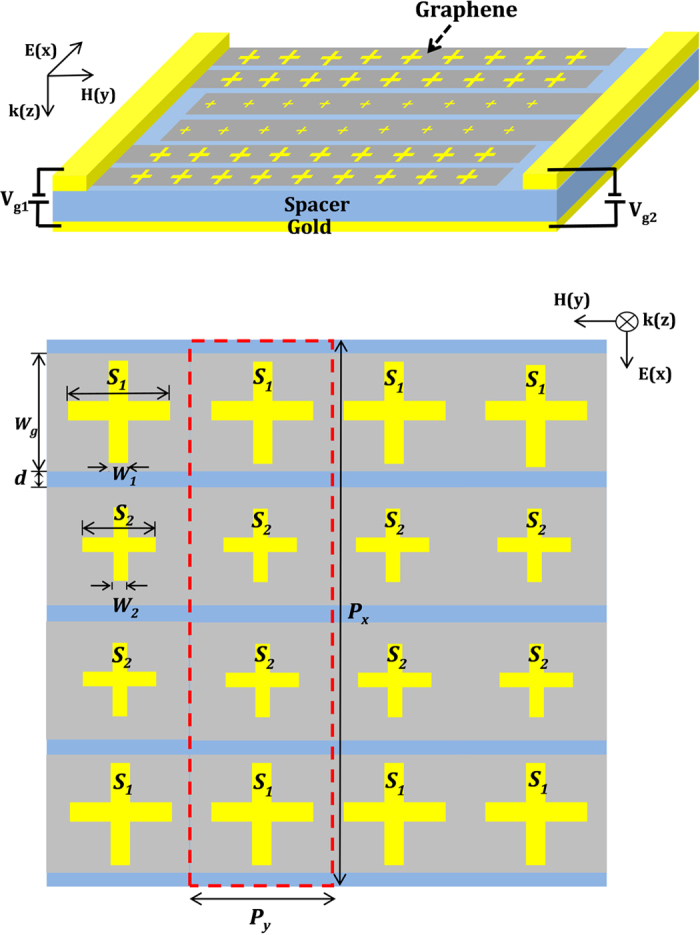
(**a**) Schematic of the independently tunable dual-band metamaterial perfect absorber consisting of an array of cross-shaped resonator pairs, a dielectric spacer, a gold ground plane, and interdigitated graphene fingers enabling independent voltage bias. (**b**) Top view of the metamaterial unit cells with dimensions specified. The red dashed box represents a unit cell, containing four crosses (two of each size).

The schematic design of the proposed tunable metamaterial absorber is illustrated in Fig. 1(a), and it consists of a traditional metamaterial absorber architecture with metallic resonators separated from a ground plane by a dielectric spacer. The unit cell of the dual-band absorber contains a combination of two cross-shaped metallic resonators of different sizes (S1 and S2), as shown in Fig. 1(b). They can be configured independently to enable two absorption bands at different frequencies. A monolayer of graphene is placed between the cross-shaped gold resonators and the PTFE dielectric spacer. The thickness of the PTFE dielectric spacer is *t*_*s*_ = 0.285 μm, and the thickness of gold is *t*_*m*_ = 0.1 μm. The periods along the *x* and *y* directions are *P*_*x*_ = 14.4 μm and *P*_*y*_ = 3.6 μm, the lengths of the bigger and smaller gold crosses are *S*_*1*_ = 3.0 μm and *S*_*2*_ = 2.0 μm, and their widths are *W*_*1*_ = 0.72 μm and *W*_*2*_ = 0.6 μm, respectively. The permittivity of the PTFE spacer is 2. The graphene layer is structured into interdigitated fingers with width *W*_*g*_ = 3.4 μm and spacing *d* = 0.2 μm. Two gate voltages, *V*_*g1*_ and *V*_*g2*_, are applied to the two graphene interdigitated finger sets in order to tune their Fermi energies independently.

The simulations were carried out using the well-established three-dimensional full wave electromagnetic solver, CST Microwave Studio 2012. We obtained the S parameters (*S*_*21*_ for transmission and *S*_*11*_ for reflection) and the absorbance was derived using 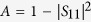, as the transmission is zero due to the thick gold ground plane.

**Figure 2:**
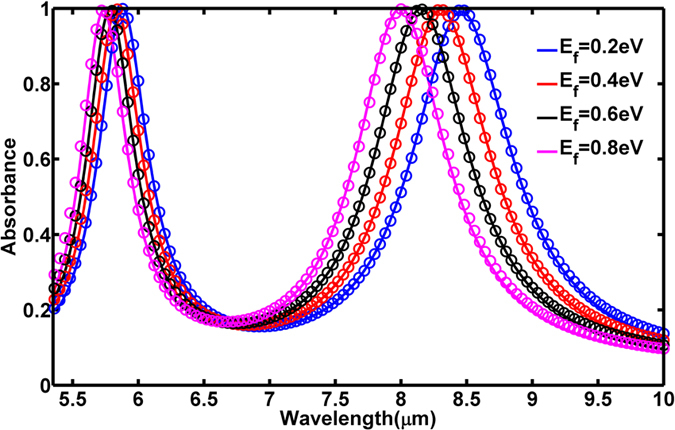
Simulated dual-band absorbance spectra (solid curves) and absorbance in circuit model (circles) at various graphene Fermi energy *E*_*f*_.

We first investigated a dual-band metamaterial absorber shown in Fig. 1 except that the graphene layer is uniform over the entire sample. The simulated absorbance spectra are plotted as the solid curves in Fig. 2 for various values of Fermi energy. Within the limit of our considered wavelength, the conductivity of the graphene layer is dominated by the intraband conductivity which can be modeled using Drude formula. The conductivity of the graphene was estimated using the calculated plasma frequency (*ω*_*p*_) and the scattering rate (*Γ*) for various Fermi energy as explained in Methods. By optimizing the thickness of the spacer *t*_*s*_ = 0.285 μm, we obtained two absorption bands with peaks absorbance greater than 99%. When *E*_*f*_ = 0.2 eV, the two absorption peaks are positioned at wavelengths of 5.88 μm and 8.46 μm. Varying *E*_*f*_ from 0.2 eV to 0.8 eV, which could be accomplished by applying a voltage bias between the ground plane and graphene layer, both absorption peaks show blue-shift while the high absorbance is remained. The longer wavelength absorption peak shifts by Δ*λ*_*S1*_ = 0.46 μm (5.4% shift), and the shorter wavelength absorption peak shifts by Δ*λ*_*S2*_ = 0.15 μm (2.6% shift). The difference in the absorption wavelength shift is mostly due to the wavelength dependent change of the imaginary conductivity 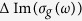, which is larger at longer wavelengths^44^, a direct consequence of the Drude-like graphene conductivity.

**Figure 3:**
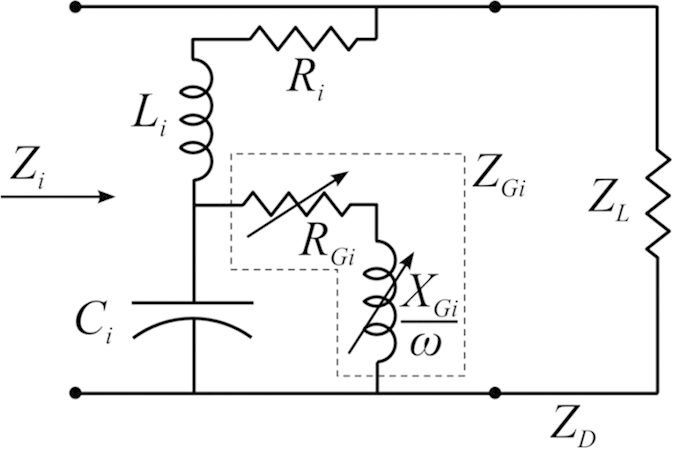
Circuit model employed in the calculation of *H*_*i*_ where *i* = (1, 2) represent resonators CR1 and CR2, respectively.

In order to further understand the blue-shift, we describe the metamaterial absorber as a combination of two independent transfer functions, *H*_*1*_ and *H*_*2*_, which apply to the corresponding two resonant structures. The wave reflected from the metamaterial absorber is quantified by


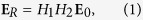


where **E**_0_ is the normally incident electric field. The absorbance is then given by 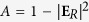. Each transfer function is calculated by a circuit model, where the cross-shaped resonator is modeled as an RLC circuit shunting a TEM transmission line, as shown in Fig. 3. The graphene layer is modeled by a separate shunt impedance *Z*_*G*_ across the capacitor *C*. The entire circuit is backed by a transmission line of impedance 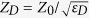 and a single load resistor, *Z*_*L*_, representing the gold ground plane. The length of transmission line *Z*_*D*_ is equal to the dielectric spacer thickness employed in the simulations and has the same effect of creating a resonant cavity between *Z*_L_ and the RLC circuit^45^. This, in combination with the complex impedance of the RLC circuit, enables nearly perfect absorption.

The circuit presents a complex, frequency-dependent impedance *Z*_*i*_ to the wave incident from a transmission line of impedance *Z*_*0*_ = 377 Ω. The transfer functions are equivalent to field reflection coefficients


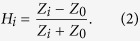


The load impedance *Z*_*L*_ = 2 Ω was calculated using tabulated bulk gold conductivity values^46^ and assuming a wave penetration depth of about 3 skin depths (3δ_d_~100 nm). The graphene impedance consists of real and imaginary parts,


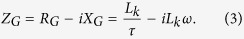


The term *L*_*k*_ = 1/α is the kinetic inductance of the graphene and, being variable with  *E*_*f*_, is the tuning mechanism of the absorber. By the equation for *Z*_*G*_ it should be hypothesized that because 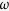 ≫ 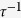 where 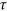 is the relaxation time, at short wavelengths the resistive part of *Z*_*G*_ should play a minor role. However, this should not hold true at longer wavelengths and *R_G_* should begin to have a noticeable effect.

The values for *R*_*i*_, *L*_*i*_, and *C*_*i*_ were determined empirically by matching the resonance frequency, Q-factor, and peak absorbance qualities of both resonances against simulations not involving any graphene layer. It was found that these factors were highly constrained if all three qualities were to be simultaneously matched. Numerical values for *Z*_*Gi*_ were calculated as


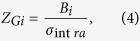


where *B*_*i*_ is a constant fitting parameter that is easily obtained by matching the magnitude of the frequency shift of the absorption peak over *E*_*f*_ = [0.2 : 0.8] eV. After *Z*_*Gi*_ was determined, the factors *R*_*i*_, *L*_*i*_, and *C*_*i*_ were fine-tuned to give the final model parameters. These are summarized in Table 1.

The equivalent circuit model results are summarized as the circles in Fig. 2. As shown, the model behaves almost exactly as the simulation in every important aspect, including the magnitude of the absorption shift, the maintenance of the absorption peak, and the maintenance of the absorption bandwidth. Importantly, all of these qualities are matched during tuning by changes in the graphene conductivity alone. Several important implications can be drawn from the model, and are not obvious from the simulations. The first is that the two structures behave entirely independent of one another, despite their interdigitated and close patterning. This fact is revealed in the independent treatment of each periodic structure as a separate transfer function. The second is that the graphene predominately alters the capacitive reactance of the structures during tuning. Alternative models, where *Z*_*G*_ shunted the entire RLC circuit or the LC sub-circuit, were also studied. In these cases, the absorbance bandwidth and/or the peak absorbance could not be kept constant in tuning over the entire Fermi energy range. By restricting the graphene effect to the capacitor alone, the net inductive reactance of *Z*_*i*_ is largely maintained during tuning, being dominated by *L*_*i*_. Though the effective capacitive reactance changes, it has very little effect on the Q-factor. Thus, absorption bandwidth is almost completely unaffected during tuning. The net resistive impedance of *Z*_*i*_ is also maintained, being dominated almost entirely by *R*_*i*_ for all values of *E*_*f*_. This prevents any change in the peak absorbance during tuning. The third implication is the validation of the hypothesis that *R*_*G*_ plays a required role in the tuning behavior at long wavelengths. By artificially zeroing *R*_*G*_ in the circuit model, a negligible effect was observed on the CR2 resonant absorbance, whereas the CR1 resonant absorbance peak continually decayed during tuning.

**Figure 4:**
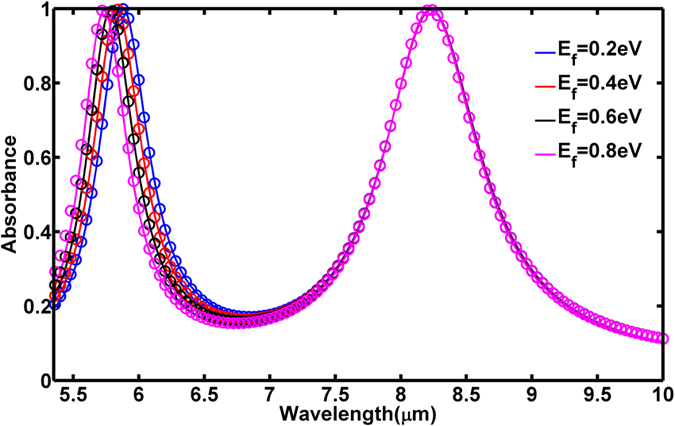
Simulated dual-band absorbance spectra (solid curves) and absorbance spectra in circuit model (circles) tuned by Fermi energy of graphene under the smaller cross-shape resonators (CR2).

To investigate the independent tuning of each resonance, we adopt the device design shown in Fig. 1(a), where the graphene layer is structured in interdigitated fingers. Every pair of fingers of graphene contains metallic resonators of the same size. These alternate finger pairs are connected at the far ends using metallic bus-lines; therefore, all bigger resonators (CR1) are electrically separated from smaller resonators (CR2). The design allows tuning the Fermi energy of graphene layers under bigger and smaller resonators independently using bias *V*_*g1*_ and *V*_*g2*_, respectively. First, we calculated the absorbance spectrum of our absorber by changing the Fermi energy of graphene under the small resonators (CR2) via tuning bias voltage *V*_*g2*_, while keeping the Fermi energy of graphene under the bigger resonators (CR1) fixed to be 0.5 eV. The simulated absorbance spectra are shown as the solid curves in Fig. 4, having two strong absorption peaks for each particular Fermi energy. As the Fermi energy of graphene under the small resonators increases from 0.2 eV to 0.8 eV, the resonant frequency related to CR2 resonator blue-shifts from *λ*_*2*_ = 5.88 μm to *λ*_*2*_ = 5.73 μm, a wavelength shift of 2.6%. An alternative expression of modulation is obtained by fixing the observation wavelength to *λ*_*2*_ = 5.88 μm. In this case one would observe an absorbance change 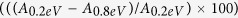 of 29% during tuning. The Fermi energy of the graphene layer under the CR1 resonators is fixed, so the resonance at *λ*_*1*_ = 8.23 μm arising from the bigger resonators remains nearly invariant. This implies that the two resonances are spectrally separated enough to prevent any noticeable mutual coupling. Thus we can independently tune the resonant frequency of the CR2 resonator by changing the Fermi energy of graphene under the small crosses. The same results can be obtained from the circuit model by fixing the Fermi energy of *H*_1_ to be 0.5 eV, which are shown as the circles in Fig. 4.

**Figure 5:**
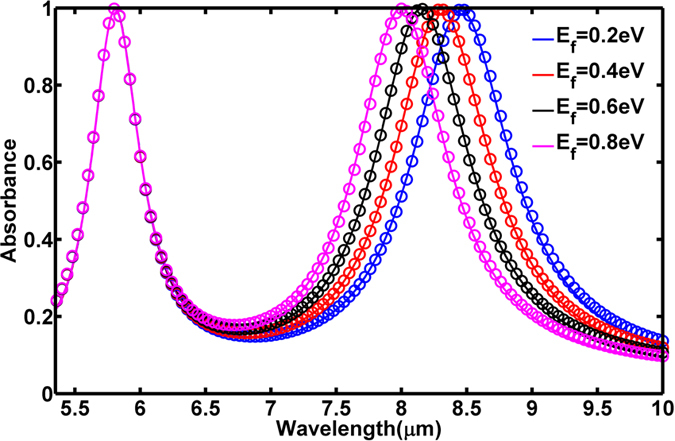
Simulated dual-band absorbance spectra (solid curves) and absorbance spectra in circuit model (circles) tuned by Fermi energy of graphene under the big cross-shape resonators (CR1).

Next, we calculated the absorption while varying the Fermi energy of the graphene layer under the bigger resonators (CR1) while fixing the Fermi energy of graphene under the smaller resonators (CR2) to 0.5 eV. The simulated absorbance spectra for various Fermi energies are shown as the solid curves in Fig. 5. As the Fermi energy of graphene under the CR1 resonator increases from 0.2 eV to 0.8 eV, its resonant wavelength *λ*_*1*_ blue-shifts from 8.45 μm to 8.0 μm, a wavelength shift of 5.3%. An alternative expression of modulation is obtained by fixing the observation wavelength to *λ*_*1*_ = 8.45 μm. In this case one would observe an absorbance change 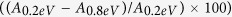 of 50% during tuning for the bigger resonator while keeping the resonant wavelength of smaller resonator fixed. The same results can be obtained from the circuit model by using the same parameters, which are shown as the circles in Fig. 5. The number of independently tunable resonators can be further increased by a multilayered approach to extend spectral coverage; however, this will require more complex design and increase fabrication difficulties.

**Figure 6:**
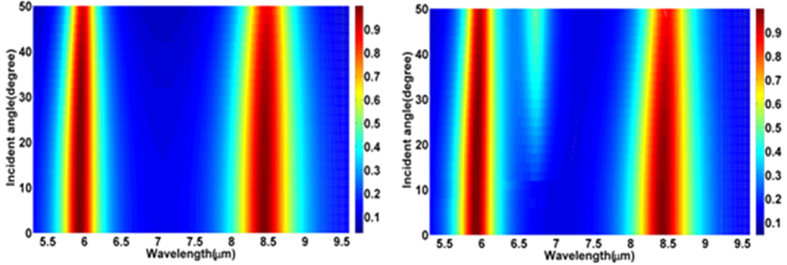
Absorbance spectrum as a function of wavelength and incident angle under (a) TE and (b) TM polarization for *E*_*f*_ = 0.4 eV.

We have conducted additional simulations to investigate the behavioral dependence on angle of incidence. For these simulations we fix the Fermi energy of graphene under both resonators to be 0.4 eV, while maintaining all other aforementioned parameters. Absorbance as a function of wavelength and incident angle is shown in Fig. 6(a,b) under TE and TM polarizations, respectively. The two red regions in each of the figures show that absorbance in both bands remains strong from 0 to 50 degrees. Nearly 90% absorbance is still obtained at large incidence angles for both of the two resonance peaks under TE and TM polarizations We have investigated the incident angle-dependent absorbance for various Fermi energies (result not shown here) and have not observed any Fermi energy related discontinuity in the absorption bands that were noticed in earlier demonstration^33^. Therefore, our design offers a better robustness for oblique incidence for both TE and TM polarizations.

**Figure 7:**
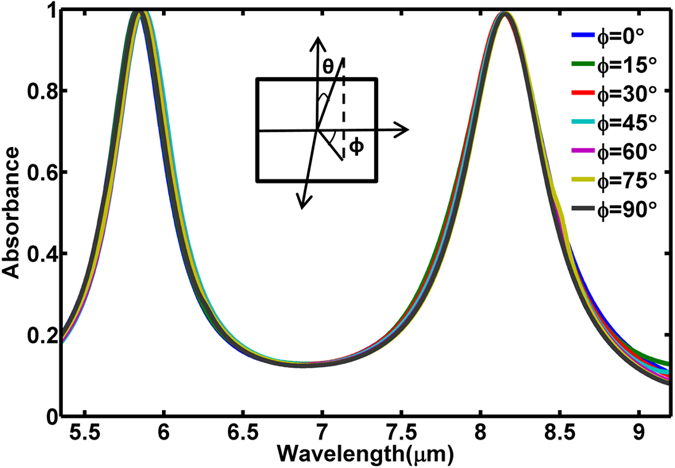
Absorbance spectra versus wavelength at different azimuthal angles φ.

It should be noted that polarization-insensitive absorbers are highly desirable^47^,^48^,^49^,^50^,^51^. To show this property, we scanned the azimuthal angle φ from 0° to 90°. The corresponding absorbance spectra are shown in Fig. 7. Here, azimuthal angle φ = 0° represents the incident electric field along x-axis and φ = 90° represents the incident electric field along y-axis. The simulated result shows that the dual-band absorbance remains nearly unchanged with the variation of azimuthal angle. Thus we can conclude that the metamaterial absorber is highly polarization independent.

We also note that the scattering rate of graphene may vary due to the impurities, defects, and interfaces^52^. To show the validity of our design, we performed numerical simulations for a similar structure by setting the graphene scattering rate 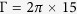 THz (a 6.2 × increase). The results (not shown here) reveal similar frequency and amplitude tuning, indicating that our design is applicable for tunable devices with realistic graphene films.

## Conclusions

In conclusion, we showed that a dual-band absorber can be formed by using horizontally cascaded cross-shaped resonant structures at mid-infrared frequencies. Absorption peaks of greater than 99% were obtained under normal incidence, and high absorbance remained at large incident angles up to 50 degrees for both of the bands under TE and TM polarizations. Moreover, our metamaterial absorber structure enabled independent tunability of the two absorption bands by electrically controlling the Fermi energy of corresponding graphene layers. With its excellent performance, our design should be applicable in many fields such as tunable sensors and selective thermal emitters.

## Methods

The simulations were carried out using commercial Finite Element full wave electromagnetic solver CST Microwave Studio 2012. We employed a time-domain solver to obtain frequency-dependent absorption spectra in Figs 2, 4 and 5 under normal incidence. The incident angle dependent absorptions in Fig. 6 were calculated using a frequency-domain solver by necessity to accommodate the oblique incidence angle. To check consistency, we compared the simulated absorptions obtained from both time- and frequency-domain at normal incidence for various Fermi energies. The results agreed very well. For the time-domain solver, we applied perfect electrical and perfect magnetic boundary conditions along the x and the y directions, respectively, with an open boundary condition in the z direction. For the frequency-domain solver, we adopted unit-cell boundary conditions in the x and y directions with Floquet ports in the z directions. The thickness of graphene layer was set to *t*_*g*_ = 0.5nm for all of our simulations. The effective dielectric constant of graphene can be calculated by 
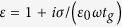
, where *t*_*g*_ is the thickness of graphene.

The complex surface conductivity of graphene can be calculated from the well-known Kubo formula and is described with interband and intraband contributions as^32^


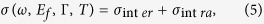



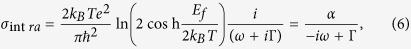



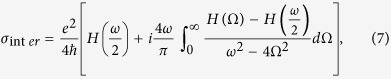


where


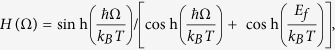


*ω* is the angular frequency, *E*_*f*_ is the Fermi energy of graphene, Γ is the collision angular frequency, *T* is the temperature, *k*_*B*_ is the Boltzmann constant, *e* is the elementary charge, and 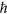 is the reduced Planck’s constant. When the Fermi energy is greater than half of the photon energy, 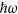 < 2*E*_*f*_, the intraband contribution dominates the graphene conductivity as the interband transitions are negligible due to Pauli blocking^53^. This condition could be approximately satisfied if we set *E*_*f*_ >0.2 eV for wavelengths of interest ranging from 5.5 μm to 10 μm. Then, graphene can be described by taking only the intraband part of the Drude-like conductivity as described in Eq. (6). We can obtain the graphene plasma frequency from


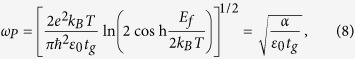


where *t*_*g*_ = 0.5 nm is the thickness of graphene layer. In our simulations, we set *T* = 300 K and scattering 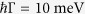 (i.e., 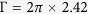 THz)^44^.

The relationship of the Fermi energy 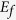 of the graphene and the voltage bias 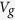 satisfy the following equation^33^


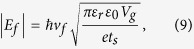


where 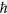 is the Planck’s constant, 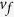 is the Fermi velocity (~1.1 × 10^6 ^m/s), 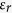 and 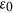 are the permittivity of the spacer and vacuum respectively. Generally, the chemical potential can be tuned over a wide range (typically from −1 eV to 1 eV)^53^,^54^,^55^. Therefore one can set the Fermi energy near 0.5 eV chemically and then tune Fermi energy from 0.2 eV to 0.6 eV by applying corresponding voltage bias of −240 V to +240 V. To avoid dielectric breakdown of the spacer we also plan to use SiO_2_ and Kapton which require lower voltage bias while offering higher dielectric breakdown for our experimental demonstration. A recent demonstration shows that the gate voltage bias can be significantly reduced by applying a layer of ionic gel over graphene film with top gating architecture^56^, which can be easily adapted to our design.

## Additional Information

**How to cite this article**: Zhang, Y. *et al.* Independently tunable dual-band perfect absorber based on graphene at mid-infrared frequencies. *Sci. Rep.* **5**, 18463; doi: 10.1038/srep18463 (2015).

## Figures and Tables

**Table 1:** Empirically determined circuit parameters to match simulations.

Structure	*R* (Ω)	*L* (pH)	*C* (aF)	*B*
CR1 (big)	12.5	0.935	16.2	0.751
CR2 (small)	31.0	2.36	3.65	3.66
